# Therapeutic effect of urine-derived stem cells for protamine/lipopolysaccharide-induced interstitial cystitis in a rat model

**DOI:** 10.1186/s13287-017-0547-9

**Published:** 2017-05-08

**Authors:** Jia Li, Hui Luo, Xingyou Dong, Qian Liu, Chao Wu, Teng Zhang, Xiaoyan Hu, Yuanyuan Zhang, Bo Song, Longkun Li

**Affiliations:** 1 Department of Urology, Second Affiliated Hospital, Third Military Medical University, Chongqing, 400037 China; 2 Department of Physical examination, Second Affiliated Hospital, Third Military University, Chongqing, 40037 China; 3 Wake Forest Institute of Regenerative Medicine, Wake Forest University, Winston Salem, North Carolina USA; 4 Department of Urology, First Affiliated Hospital, Third Military University, Chongqing, 40037 China

**Keywords:** Urine, Bladder, Stem cells, Inflammation

## Abstract

**Background:**

Interstitial cystitis (IC) is a chronic inflammation disorder mainly within the submucosal and muscular layers of the bladder. As the cause of IC remains unknown, no effective treatments are currently available. Administration of stem cell provides a potential for treatment of IC.

**Methods:**

This study was conducted using urine-derived stem cells (USCs) for protamine/lipopolysaccharide (PS/LPS)-induced interstitial cystitis in a rodent model. In total, 60 female Sprague–Dawley rats were randomized into three experimental groups (*n* = 5/group): sham controls; IC model alone; and IC animals intravenously treated with USCs (1.2 × 10^6^ suspended in 0.2 ml phosphate-buffered saline (PBS).

**Results:**

Our data showed that the bladder micturition function was significantly improved in IC animals intravenously treated with USCs compared to those in the IC model alone group. The amount of antioxidants and antiapoptotic protein biomarkers heme oxygenase (HO)-1, NAD(P)H quinine oxidoreductase (NQO)-1, and Bcl-2 within the bladder tissues were significantly higher in IC animals intravenously treated with USCs and lower in the sham controls group as assessed by Western blot and immunofluorescent staining. In addition, the expression of autophagy-related protein LC3A was significantly higher in the IC model alone group than that in IC animals intravenously treated with USCs. Inflammatory biomarkers and apoptotic biomarkers (interleukin (IL)-6, tumor necrosis factor (TNF)α, nuclear factor (NF)-κB, caspase 3, and Bax) and the downstream inflammatory and oxidative stress biomarkers (endoplasmic reticulum stress and autophagy-related protein (GRP78, LC3, Beclin1)) in the bladder tissue revealed statistically different results between groups.

**Conclusions:**

USCs restored the bladder function and histological construction via suppressing oxidative stress, inflammatory reaction, and apoptotic processes in a PS/LPS-induced IC rodent model, which provides potential for treatment of patients with IC.

## Background

Bladder pain syndrome/interstitial cystitis (BPS/IC) has the characteristics of bladder pain plus the urinary symptoms of urgency, frequency, and nocturia [1]. In the nineteenth century, it was first described by the presence of red, bleeding areas on the bladder wall, known as Hunner’s lesions [2]. It is a chronic, noninfectious, probably inflammatory disorder of the urinary bladder [3]. The morbidity of BPS/IC ranges from 1 in 100,000 to 5.1 in 1000 in the worldwide population [3–5], and approximately 90% of patients are women [6]. BPS/IC reduces the quality of life for 3.3–7.9 million people in the US alone [2]. Despite the prevalence and highly investigated etiology and mechanisms of the obstinate disease, there have been only a few effective treatments reported so far. The key event for IC will be to find a safe and efficacious treatment.

Stem cell therapies have become the latest strategy aimed at treating IC. Human mesenchymal stem cells (MSCs) successfully alleviated IC in a rat model in which the Wnt pathways stimulated the regeneration of the damaged bladder epithelium [7]. Combined melatonin and adipose-derived MSC (ADMSC) treatment offered an additional benefit compared with monotherapy in protecting the urinary bladder against cyclophosphamide-induced acute IC in rats through suppression of oxidative stress and the inflammatory reaction [8]. However, the process to obtain these stem cells is very complicated. Therefore, a more concise and efficient strategy in stem cell therapy for IC is necessary.

Tissue-specific stem cells, such as ADMSCs [9], embryonic stem cells [10], and muscle-derived stem cells [11], are a very small subpopulation of cells, and isolating them from differentiated somatic cells present in the tissues and organs is very difficult. However, urine-derived stem cells (USCs), a subpopulation of stem cells that display many characteristics of MSCs, can be easily isolated from human voided urine [12–15]. USCs have the capacity for multipotent differentiation including endothelial and smooth muscle cells [16]. Therefore, USCs may represent a more promising cell therapy for the treatment of urinary dysfunction. One study clarified that human USCs or USCs genetically modified with fibroblast growth factor (FGF)2 revealed a more efficient therapeutic effect in erectile dysfunction (ED) in rats than MSCs [17]. Based on previous findings, we sought to test the hypothesis that USCs could protect against protamine/lipopolysaccharide (PS/LPS)-induced IC in a rat model through suppression of oxidative stress, the inflammatory reaction, and apoptosis.

## Methods

### Study design

Sixty female Sprague–Dawley (SD) rats were used in our study, which was approved by the Research Council and Animal Care and Use Committee of the Third Military Medical University, China. A total of 60 female SD rats weighing 200–230 g were housed at 20–25 °C under a standard 12 h/12 h light–dark cycle, and all rats received a standard diet. They were randomized into three experimental groups (20 rats each): sham controls, IC alone, and IC + USCs. Five female SD rats from each group was tested for 24-h urine volume level and Western blot detection of inflammatory biomarkers (interleukin (IL)-6, tumor necrosis factor (TNF)α, and nuclear factor (NF)-κB), apoptotic biomarkers (caspase 3 and Bax), endoplasmic reticulum stress (ERS) and autophagy-related proteins (GRP78, LC3A/B, and Beclin1), antioxidant biomarkers (heme oxygenase (HO)-1 and NAD(P)H quinine oxidoreductase (NQO)-1), and an antiapoptotic biomarker (Bcl-2). A group of five female SD rats was sacrificed for immunofluorescence (IF) testing for antioxidant biomarkers (HO-1 and NQO-1), immunohistochemical (IHC) testing for an autophagy biomarker (LC3), and the other 5 rats for histological testing. The remaining five female SD rats from each group were prepared for cystometry assessments.

### USC culture in vitro and the experimental model of IC

We collected mid- and last-stream urine samples (average approximately 200 mL/sample) from five human healthy male individuals who were 25 to 29 years old. We centrifuged each urine sample at 500 × g for 5 min to collect cells. The supernatant was carefully aspirated, and the cell pellet was gently resuspended in the initiation medium, which was a 1:1 mixture of KSFM and embryonic fibroblast medium (EFM) [15, 18]. The medium was changed every other day, and cells were split at 70–80% confluence as we previously reported [12].

**Fig. 1:**
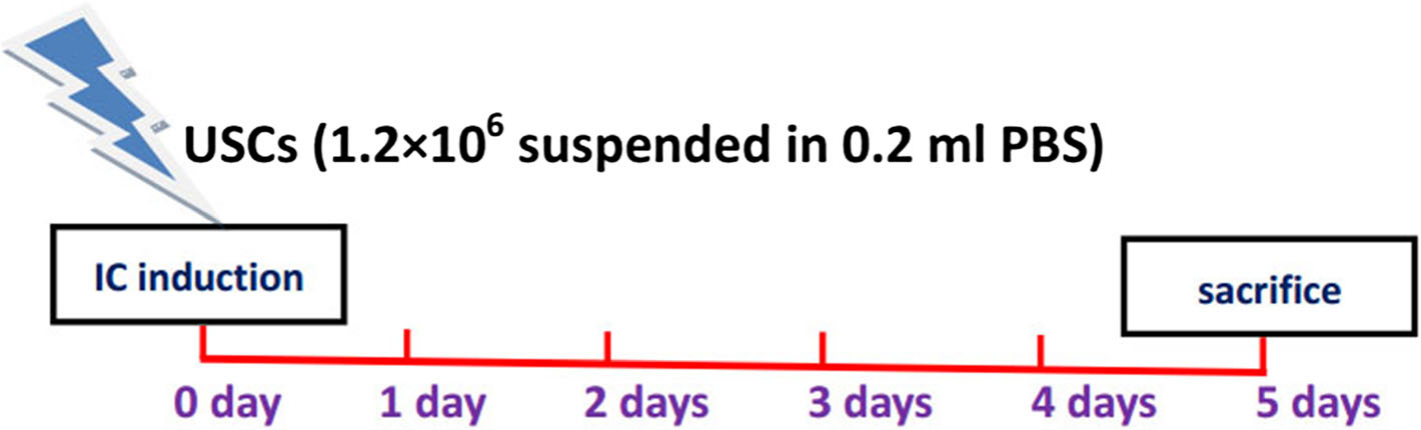
Timeline showed the induction of interstitial cystitis (*IC*), injection of urine-derived stem cells (*USCs*), and time point of sacrifice. *PBS*, phosphate-buffered saline

The IC animal model received 10 mg/mL PS in the urinary bladder, 2 mg/mL LPS 30 min later, and the experimental conditions 45 min later as previously described [19]. The sham control group received a saline instillation only. The IC + USC group was given USCs (1.2 × 10^6^ cells suspended in 0.2 ml phosphate-buffered saline (PBS)) intravenously after the IC model was generated. The animals were sacrificed with high-dose anesthesia 5 days after treatment, and their bladders were removed (Fig. 1).

### Collecting 24-h urine for quantification of urine volume and hematuria assessment at 4 days after IC induction

Twenty-four-hour urine samples were collected using metabolic cages from five female SD rats from each group at 4 days after IC induction prior to sacrificing the animals to test the color and volume of the urine and for hematuria in a clinical laboratory in Xinqiao Hospital.

### Quantitative analysis of inflammatory, antiinflammatory, oxidative stress, antioxidative stress, apoptotic, antiapoptotic, ERS, and autophagy biomarkers in the bladder using Western blot

The bladder wall (*n* = 5) in each group was used for Western blotting. The bladder tissue was cut into small pieces, homogenized, and dissociated in radio-immunoprecipitation assay lysis buffer at 4 °C for 30 min. The homogenate was centrifuged at 15,000 × g for 10 min at 4 °C, and the supernatant was collected and used as the total protein. The protein concentration was measured. Following electrophoresis, we used a polyvinylidene difluoride (PVDF) membrane to transfer the proteins. Nonspecific proteins were blocked by incubating the membrane in 5% nonfat dry milk in T-TBS containing 0.05% Tween for 2 h. The membranes were incubated overnight with primary antibodies, including the rabbit monoclonal antibodies anti-NQO1 (1:1000; Abcam, ab80588), anti-HO1 (1:1000; Abcam, ab68477), anti-LC3A/B (1:1000; Cell Signaling Technology, #12741), anti-Beclin-1 (1:1000; Cell Signaling Technology, #3495), and anti-NF-κB (1:1000; Cell Signaling Technology, #8242). The rabbit polyclonal antibodies included anti-GRP78 (1:1000; Abcam, ab21685), anti-Bax (1:1000; GeneTex, GTX109683), anti-Caspase3 (1:1000; GeneTex, GTX110543), anti-Bcl2 (1:1000; GeneTex, GTX100064), anti-IL-6 (1:1000; GeneTex, GTX110527), anti-TNFα (1:1000; GeneTex, GTX116059), and mouse polyclonal anti-GAPDH (1:1000; Beyotime, AG019-1). The secondary antibodies were horseradish peroxidase-conjugated goat anti-mouse IgG (1:5000; Zhongshan Inc., ZB2305) and goat anti-rabbit IgG (1:5000; Zhongshan Inc., ZB-2301). The samples were incubated in 5% nonfat dry milk in T-TBS containing 0.05% Tween. We collected the protein band images and analyzed the relative optical density (ROD) with molecular imaging using a ChemiDoc XRS + Image System (Bio-Rad Laboratories, Hercules, CA, USA).

### Immunohistochemical and immunofluorescent studies

The right bladder walls of five SD rats of each group were sampled for IHC staining. First, we treated rehydrated paraffin sections with 3% H_2_O_2_ for 30 min and incubated them with ImmunoBlock reagent for 30 min at room temperature. The sections were then incubated with primary antibodies specifically against LC3A/B (1:100; Cell Signaling Technology, #12741) at 4 °C overnight. An IHC-based scoring system was used for the semiquantitative analyses of LC3A/B as a percentage of positive cells in a blind fashion (scores of positively stained cell for LC3A/B: 0 = negative staining percentage; 1 = <15%; 2 = 15–25%; 3 = 25–50%; 4 = 50–75%; 5 = 75–100% per high-power field (200 ×)).

For IF staining, the left bladder walls of five SD rats in each group were sectioned at 6 μm per slice and fixed with 4% paraformaldehyde at 4 °C for 15 min. The samples were washed in deionized water for 10 min and then incubated in PBS for 5 min. The specimens were incubated in the primary rabbit monoclonal antibodies anti-NQO1 (1:300; Abcam, ab80588) and anti-HO1 (1:300; Abcam, ab68477) at 4 °C overnight. Then, the secondary fluorescent Alexa-Fluor-488-conjugated goat anti-mouse IgG (1:100; Boster Inc., BA-1101) antibody was incubated at room temperature in the dark for 2 h. Subsequently, samples were washed three times with PBS and were identified with nuclear labeling by incubating in 4′,6-diamidino-2-phenylindole (DAPI) for 5 min at room temperature. Finally, the specimens were observed under a confocal microscope. Three randomly selected fields were analyzed for quantification in each section of three groups. The percentage of positively stained cells per slice for each animal was then determined.

### Histological evaluation

The bladders of five SD rats from each group were transected and divided longitudinally into two sections for fixation in 4% paraformaldehyde. Five-micrometer sections were stained with hematoxylin and eosin and toluidine blue (Shengong Biotech, TE847). Subsequently, the histological score and mast cell counts were determined by an investigator in a blinded fashion. Bladder inflammation was assessed using a six-point scoring system as previously reported [21]. The quantification of mastocytes in the lamina propria and muscle layer was estimated at 200× magnification in five random sections from each group.

### TUNEL staining

A TUNEL staining assay kit purchased from Roche Applied Science for the detection of apoptosis was used on paraffin sections of the bladder of each group according to the manufacturer’s instructions. Quantitation of the number of positive cells was carried out at a magnification of 200× in five randomly chosen fields of view on each slide. The apoptosis index was calculated using the ratio of the apoptotic cells to total cells.

### Bladder cystometry

Under urethane anesthesia, five SD rats from each group had body temperature (37–38 °C) saline infused at a rate of 0.1 mL/min using a syringe pump followed by the placement of a PE-50 catheter into the bladder. After a stabilizing time of approximately 30 min, urodynamic parameters including basal pressure (BP), maximum pressure (MP), and micturition frequency (MF) were evaluated.

### Statistical analysis

SPSS v13.0 (SPSS) was used for all analyses by an investigator blinded to the treatment groups. A one-way analysis of variance (ANOVA) was used to analyze all outcomes, followed by Fisher’s test to assess differences among treatment groups. A probability value of <0.05 was considered significant.

## Results

### Changes in urine appearance, hematuria, and urine volume

**Fig. 2:**
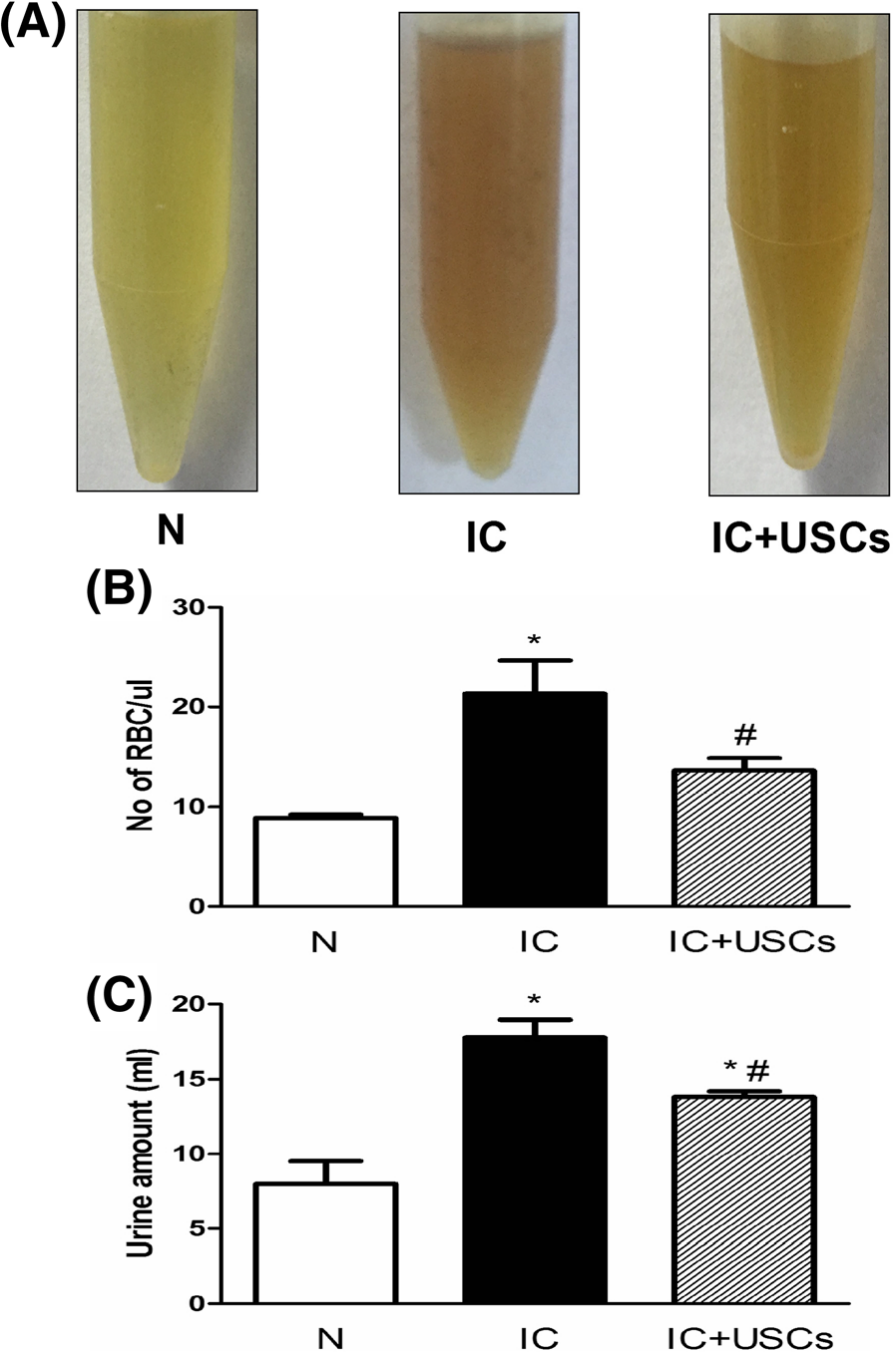
Urine appearance, volume, and hematuria after protamine/lipopolysaccharide-induced interstitial cystitis (*n* = 5). **a** Urine appearance of the interstitial cystitis (*IC*) group was most turbid by visual observation. The urine of the IC + urine-derived stem cell (*USC*) group was remarkably more turbid than that of the control group (*N*) and less turbid than that of the IC group at 4 days after IC induction. **b** The number of red blood cells (*RBC*)/μL in the urine. **c** Twenty-four-hour urine volume by the end of 4 days in all groups. Data are expressed as the mean ± SD. *P < 0.01, versus the control group; ^#^ *P* < 0.05, versus the IC group

In the IC group, the urine appearance was most turbid. The urine of the IC + USC group was less turbid than the urine of the IC group yet more turbid than the control at 4 days after IC induction (Fig. 2a). Additionally, the indicator of hematuria (the number of red blood cells/μL) revealed that the highest number was in the IC group, with more than 20 cells/μL (*P* < 0.01). In the IC + USC group, the red blood cell number (10–20 cells/μL) was higher than the control group (less than 10 cells/μL) and lower than the IC group (Fig. 2b) (*P* <0.05). The 24-h urine volume showed the same pattern as the number of red blood cells (Fig. 2c).

### Evaluation of inflammation-related factors

**Fig. 3:**
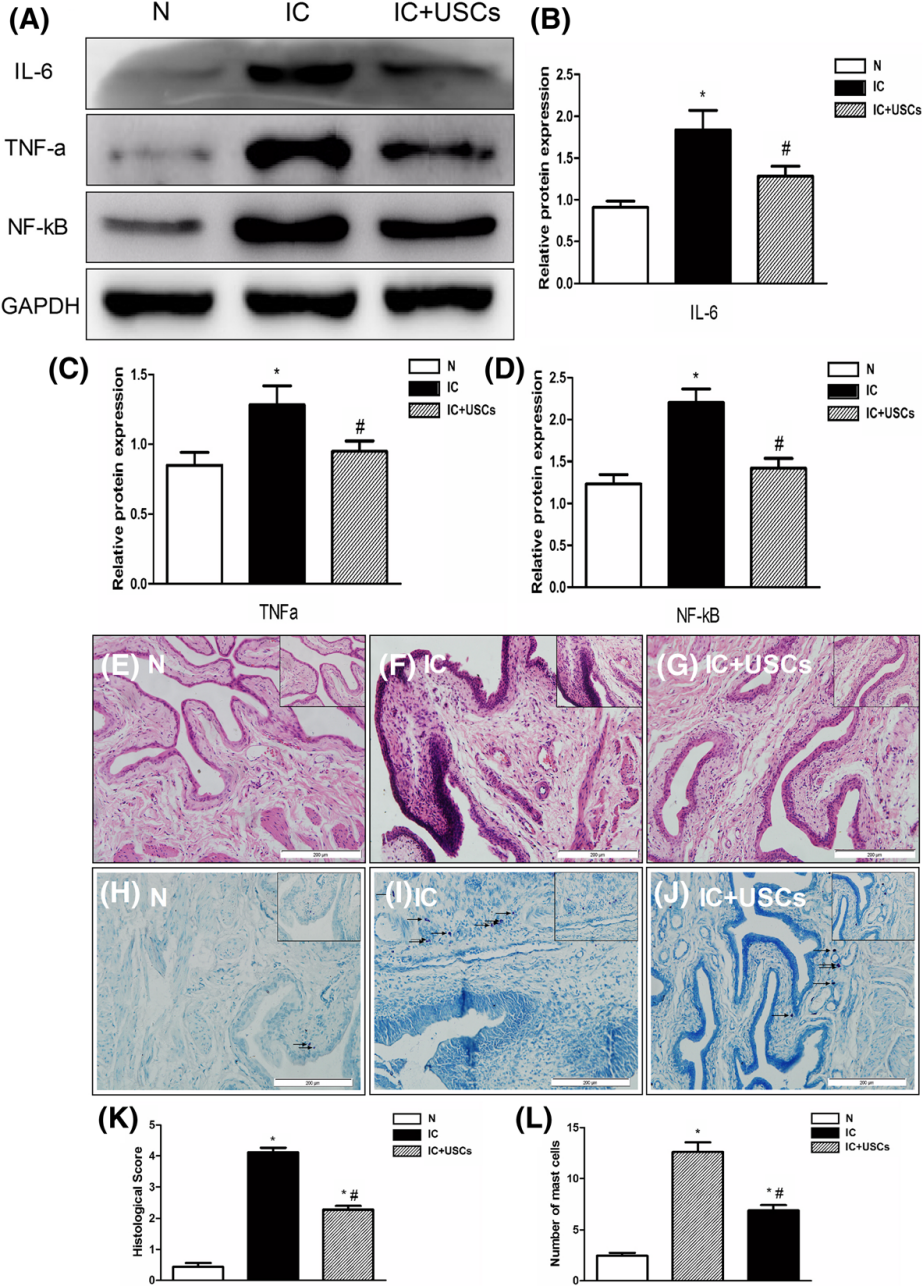
Expression of inflammatory-related factors in the urinary bladder at 4 days after IC induction (*n* = 5). **a** Expression of the inflammatory biomarkers interleukin-6 (*IL-6*), tumor necrosis factor alpha (*TNF*α), and nuclear factor kappa B (*NF-*κ*B*) by Western blot in the bladder tissue was highest in the interstitial cystitis (*IC*) group compared to the IC + urine-derived stem cell (*USC*) group and normal control (*N*) groups; the biomarker expression in the IC + USC group was lower than in the IC group. **b** A statistical chart of the relative optical density of IL-6/GAPDH in each group (*n* = 5). **c** A statistical chart of the relative optical density of TNFα/GAPDH in each group (*n* = 5). **d** A statistical chart of relative optical density of NF-κB/GAPDH in each group (*n* = 5). **e**–**g** Photomicrograph images of hematoxylin and eosin staining in rat bladder samples (*scale bars* = 200 μm). **h**–**j** Representative photomicrograph images of rat bladder samples stained with toluidine blue (*arrows*) demonstrate mast cells (*scale bars* = 200 μm). **k** A statistical chart demonstrates the inflammation grading (*n* = 5). **l** A statistical chart reveals the number of mast cells in the bladder of rats (*n* = 5). *P < 0.05, versus the control group; ^#^ *P* < 0.05, versus the IC group

The expression of the inflammatory biomarkers IL-6, TNFα, and NF-κB by Western blot in the bladder tissue was highest in the IC group compared to the IC + USC group and the control group. The biomarker expression in the IC + USC group was lower than in the IC group (Fig. 3a–d). The bladder tissue from the IC group indicated massive ulcers, obvious edema and hemorrhage, and increased inflammatory cell infiltration (particularly mast cells) in the submucosal and muscular layer compared with the control group. This situation was significantly improved in the IC + USC group (Fig. 3e–j). The quantitative assessment of the histological score was highest in the IC group, while the score of the IC + USC group was higher than control group (*P* < 0.05) and lower than the IC group (*P* < 0.05). Mast cell count in the IC + USC group was lower than in the IC group (*P* < 0.05) and higher than in the control group (*P* < 0.05) (Fig. 3k and l). These results demonstrated that the inflammatory response was the most severe in the IC group. The assessment of the IC + USC group was more severe than the control group and alleviated compared with the IC group.

### Assessment of oxidative stress-related factors

**Fig. 4:**
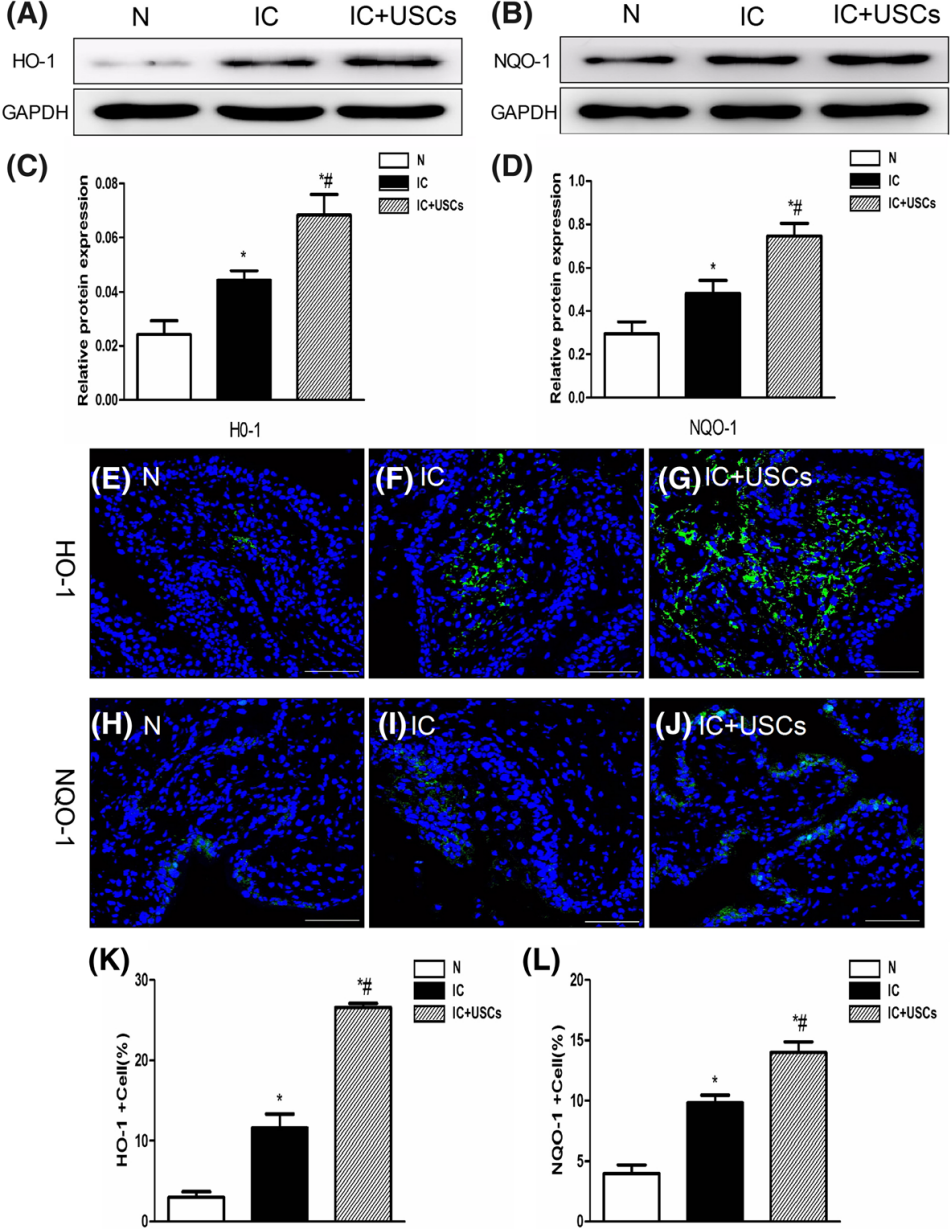
Western blot and immunofluorescent (IF) staining for the antioxidative markers HO-1- and NQO-1-positive cells in urinary bladders at 4 days after IC induction (*n* = 5). **a**,**b** The protein expression of heme oxygenase-1 (*HO-1*) and NAD(P)H quinine oxidoreductase (*NQO-1*) by Western blot was remarkably lower in the control group (*N*) than in the interstitial cystitis (*IC*) and IC + urine-derived stem cell (*USC*) groups, and notably lower in the IC group compared to the IC + USC group. **c** A statistical chart of the relative optical density of HO-1/GAPDH in each group (*n* = 5). **d** A statistical chart of the relative optical density of NQO-1/GAPDH in each group (*n* = 5). **e**–**g** Microscopic (400×) IF staining for the number of HO-1-positive cells infiltrated into the bladder in all groups. **h**–**j** Microscopic (400×) IF staining for the cellular expression of NQO-1 in the urinary bladder in the three groups of animals. **k** A statistical chart reveals the index of HO-1-positive cells in bladder tissue (*n* = 5). **l** A statistical chart reveals the index of NQO-1-positive cells in the three groups of bladder tissue (*n* = 5). *P < 0.05, versus the control group; ^#^ *P* < 0.05, versus the IC group

The protein expression of two antioxidative indicators, HO-1 and NQO-1, by Western blot was remarkably lower in the control group than in the IC group and the IC + USC group, and notably lower in the IC group than in the IC + USC group (Fig. 4a–d). Moreover, the assessment of HO-1 and NQO-1 expression by IF showed a similar pattern compared to that of the protein expression among the three groups (Fig. 4e–l). These findings suggest an antioxidative response after the induction of IC injury and an enhancement of the antioxidant effect following USC administration.

### Protein expression of apoptotic mediators

**Fig. 5:**
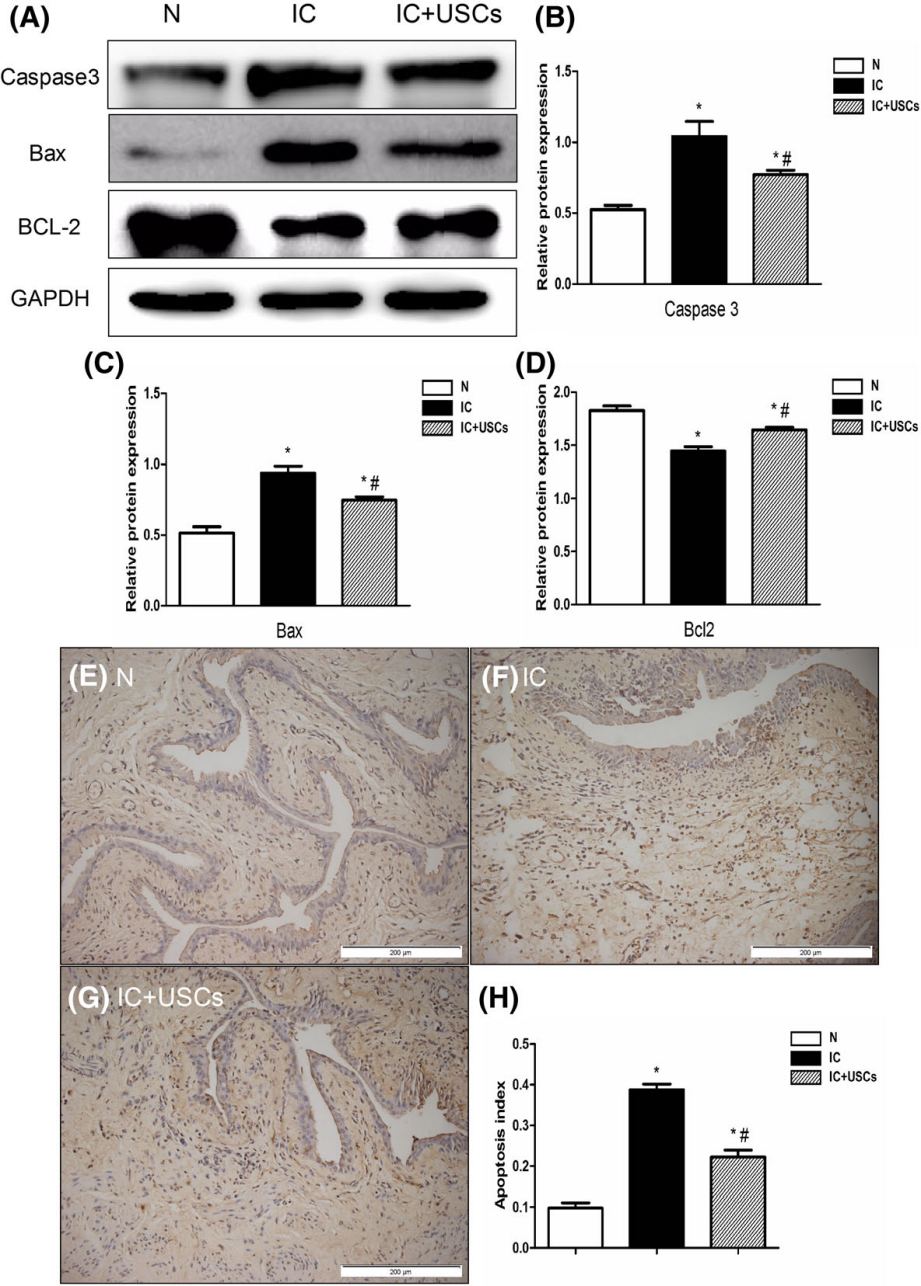
Analysis of protein expression of the apoptosis biomarkers caspase 3 and Bax and the antiapoptosis indicator BCL-2 and a TUNEL assay in the urinary bladder at 4 days after IC induction (*n* = 5). **a** Expression of the apoptosis biomarkers caspase 3 and Bax and the antiapoptosis indicator BCL-2 by Western blot in the bladder tissue was highest in the interstitial cystitis (*IC*) group compared to the IC + urine-derived stem cell (*USC*) group and normal control (*N*) group; the biomarker expression in the IC + USC group was lower than in the IC group. **b** A statistical chart of the relative optical density of caspase 3/GAPDH in each group (*n* = 5). **c** A statistical chart of the relative optical density of Bax/GAPDH in each group (n = 5). **d** A statistical chart of the relative optical density of BCL-2/GAPDH in each group (*n* = 5). **e**–**g** The TUNEL assay indicated that, in sections from the IC group rats, the most apoptotic nuclei were observed, whereas fewer were present in the IC + USC group and more were present than in the normal control group. **h** A statistical chart reveals the index of apoptotic nuclei in all groups of bladder tissue (*n* = 5). *P < 0.05, versus the control group; ^#^ *P* < 0.05, versus the IC group

The protein expression of caspase 3 and Bax, two indexes of apoptosis, were significantly higher in the IC group than in the control group and the IC + USC group, and were markedly higher in the IC + USC group than in the control group by Western blot. However, the index of antiapoptosis (Bcl-2 protein expression) was remarkably lower in the IC + USC group compared to the control group (*P* < 0.05) and higher than in the IC group (*P* < 0.05) (Fig. 5a–d). Furthermore, a TUNEL assay was performed to examine the levels of apoptosis in each group. In sections from the IC group rats, the most apoptotic nuclei were observed, whereas fewer were present in the IC + USC group and more were present in the control group (Fig. 5e–h). These results reveal that USC treatment exerted antiapoptotic effects.

### Confirmation of the differences in ERS and autophagy-related factors

**Fig. 6:**
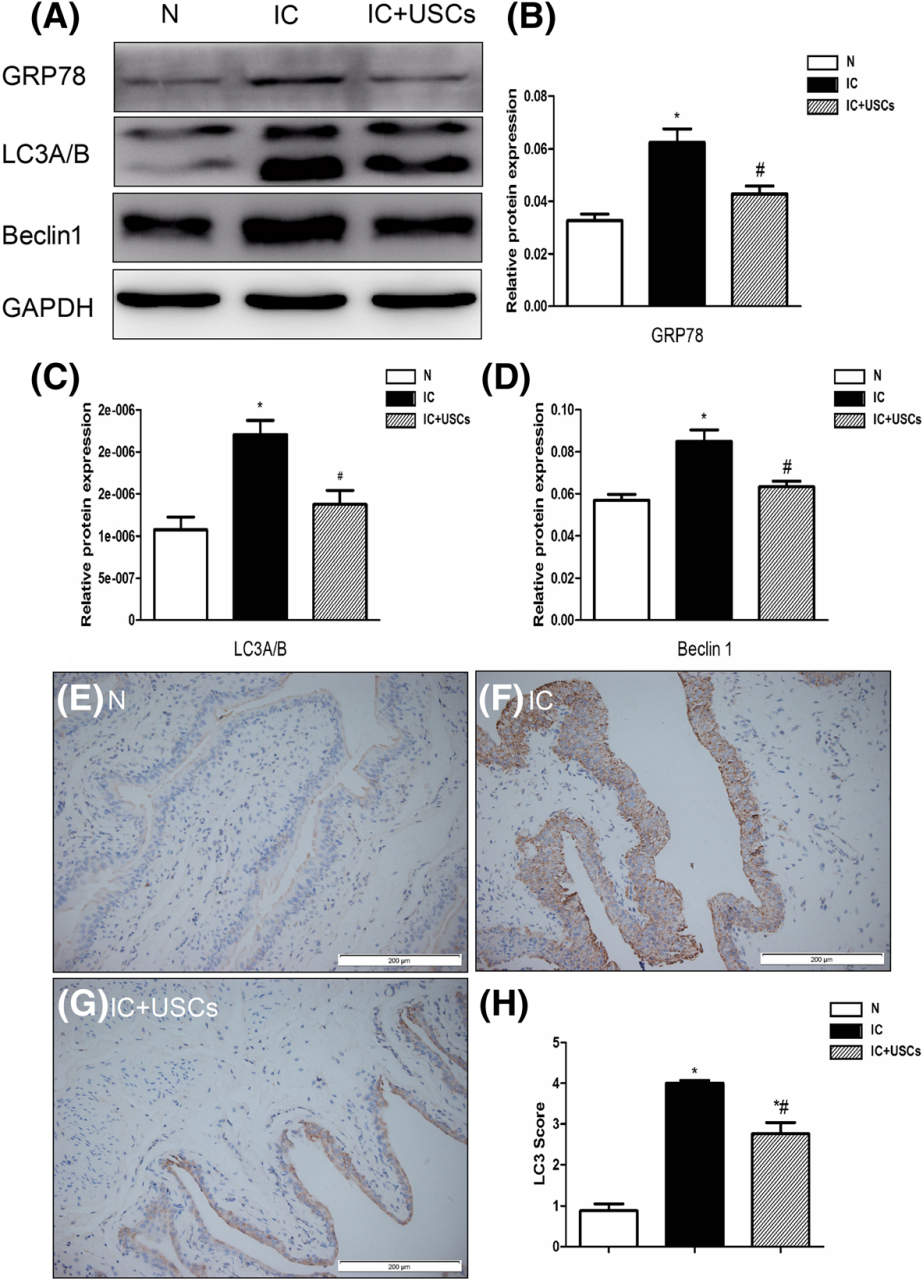
Expression of the ERS biomarker GRP78 and autophagy-related factors LC3A/B and Beclin 1 and IHC for LC3A/B in the urinary bladder at 4 days after IC induction (*n* = 5). **a** The protein expression of GRP78, LC-3A/B, and Beclin1, one indicator of ERS and two indicators of autophagy, was highest in the interstitial cystitis (*IC*) group and lowest in the normal controls (*N*), and significantly lower in the IC + urine-derived stem cell (*USC*) group than in the IC group. **b** A statistical chart of the relative optical density of GRP78/GAPDH in each group (*n* = 5). **c** A statistical chart of the relative optical density of LC3B/LC3A/GAPDH in each group (*n* = 5). **d** A statistical chart of the relative optical density of Beclin1/GAPDH in each group (*n* = 5). **e**–**g** The protein expression of the index of autophagy, LC3A/B, by immunohistochemistry in the urinary bladders of each group of animals. **h** Analytical results of LC3 score. *P < 0.05, versus the control group; ^#^ *P* < 0.05, versus the IC group

Currently, using Western blotting with GRP78, LC-3A/B, and Beclin1 detects the change in ERS and autophagy. The protein expression of GRP78, LC-3A/B, and Beclin1, which are one indicator of ERS and two indicators of autophagy, were highest in the IC group and lowest in the controls, and significantly lower in the IC + USC group compared to the IC group (Fig. 6a–d). Additionally, the protein expression of the index of autophagy (LC3A/B) by immunohistochemistry displayed a pattern similar to that of the protein LC3A/B among all groups (Fig. 6e–h). These results reveal that the internal environment of the bladder was perfect.

### Changes in urodynamic parameters

**Fig. 7:**
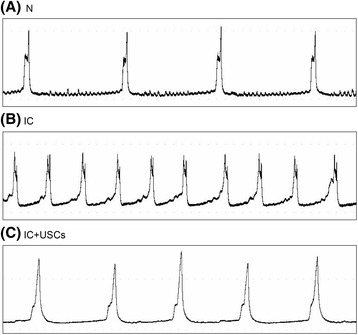
Cystometry variables of the (**a**) sham control (*N*), (**b**) interstitial cystitis (*IC*), and (**c**) IC + urine-derived stem cell (*USC*) groups

The mean bladder contractions per hour in the IC + USC group were more frequent than in the control group and slower than in the IC group. The basal pressure of the IC group was higher than in the control group, and it was reduced in the IC + USC group. No statistical significance was found in the maximum pressure compared to the control group, indicating a recovered micturition function (Table 1 and Fig. 7).

**Table 1:** Urodynamic parameters changes

Urodynamic parameters	Control group	Interstitial cystitis group	Interstitial cystitis + urine-derived stem cell group
Basal pressure (cmH_2_O)	2.10 ± 0.33	11.06 ± 5.66*	3.98 ± 2.14^#^
Maximum pressure (cmH_2_O)	54.91 ± 4.28	34.32 ± 6.51*	43.77 ± 12.59
Micturition frequency (no./h)	9.87 ± 1.00	24.20 ± 2.88*	16.63 ± 2.44*^#^

Data presented as means ± SD (*n* = 5)

*P < 0.05, versus the control group; ^#^ *P* < 0.05, versus the interstitial cystitis group

## Discussion

Urinary tissue-specific stem cells (USCs) revealed a more efficient therapeutic effect in stem cell therapy for ED in rats than mesenchymal stem cells (MSCs) [17]. Therefore, USCs may represent a more promising cell therapy for the treatment of urinary dysfunction. Hence, we conducted the current study to investigate the therapeutic impact of USCs on PS/LPS-induced interstitial cystitis in a rat model, which revealed several striking implications. First, the architectural integrity of the bladder was preserved and the deterioration of bladder function was attenuated after USC therapy according to the urine appearance and urodynamic parameters. Second, USC treatment was associated with antiinflammatory, antioxidative, and proapoptosis effects in bladder tissue after IC was induced. Third, the internal environment of the bladder tissue after IC was induced was ameliorated by USC treatment, which was indicated by reduced ERS and autophagy.

IC has the characteristics of bladder pain plus the urinary symptoms of urgency, frequency, and nocturia [24]. The 24-h urine volume and hematuria are the two typical signs of IC. Clinically, an increased urine amount might result in urinary urgency and frequency as well as nocturia. One important finding in the present study is that the 24-h urine volume, hematuria, and urination frequency were markedly aggravated in IC animals without treatment, as in previous studies [7, 8]. USC treatment offered a significant therapeutic effect in reducing urination frequency, 24-h urine volume, and the severity of hematuria in an IC rat model. Our results showed the benefit of USCs against urinary bladder injury in rodent IC and are consistent with a recent study that demonstrated significant protection offered by USCs against diabetic erectile dysfunction [17].

The inflammatory process is an important etiology of IC [22]. In patients with IC/BPS, the most common findings include bladder and pelvic pain, glomerulation under cystoscopic hydrodistention, and denudation or thinning of the bladder epithelium, suggesting that bladder inflammation and urothelial dysfunction occur in patients with IC/BPS [23, 24]. In our study, an essential finding was that the expression of the inflammatory biomarkers TNFα, NF-κB, and IL-6 was substantially augmented in IC animals without treatment compared with the control animals. We also found that the obvious edema and hemorrhage and the increased inflammatory cell infiltration (particularly mast cells) in the submucosal and muscular layer of the urinary bladder were comparable to those of previous studies [8, 19, 25]. With USC treatment, the inflammation was reduced and the architectural integrity of the bladder was preserved. These results suggested that USC treatment suppressed the inflammatory reaction in the IC rat model.

The pathogenesis of IC includes the overproduction of reactive oxygen and cytokines, which leads to extensive oxidative stress. In IC, the mechanism of oxidative damage comes into prominence [26]. In addition, in our study, we found that the antioxidant biomarkers HO-1 and NQO-1 by Western blot and IF were notably increased in the tissue of the urinary bladder in IC animals compared to those in the control group. Many stress responses such as ischemia–reperfusion injury could markedly increase these antioxidant mediators [20]. These indicators, acting as the scavengers for free radicals in the urinary bladder, were enhanced after USC treatment. These findings suggested that USC treatment suppressed oxidative stress in this experimental setting of IC injury.

Apoptosis is a stepwise process characterized by a series of stereotypical morphological changes that eventually lead to cell death [27]. The apoptotic process was highly activated in the urothelial cells of the IC/BPS specimens, which may have resulted from the upregulation of inflammatory signals [28]. In our study, when compared with the control group, the apoptotic biomarkers caspase 3 and Bax were significantly enhanced and the antiapoptotic indicator Bcl-2 was reduced in the IC group. In addition, significantly reduced expression of Bax and caspase 3 and a notably enhanced expression of Bcl-2 were demonstrated in the IC animals that received the USC treatment. Furthermore, the TUNEL assay results of the IC group rats indicated that the most apoptotic nuclei were observed, whereas fewer were present in the group that received USC treatment and more were present than in the control group. Our results showed that USC treatment protects the urinary bladder from apoptotic injury in IC rats.

In eukaryotic cells, the endoplasmic reticulum (ER) is the major site where secreted and transmembrane proteins are synthesized and folded. Depending on the physiological state and environmental conditions, the protein flux into the ER may vary substantially [29]. In IC, the physiological state and environmental conditions were changed and induced misfolded protein accumulation in the ER, which induced the unfolded protein response (UPR). The autophagy pathway and autophagy proteins may function as a central fulcrum that balance the beneficial and harmful effects of the host response to infection and other immunological stimuli [30]. IC is a type of inflammatory disease in which there is a low expression of autophagy [25]. In our study, we found that the ERS and autophagy biomarkers GRP78, LC3A/B, and Beclin1 were significantly enhanced in the IC group compared with the control group, and all of them were reduced after USC treatment. These results revealed that the physiological state, environmental conditions, and inflammation were ameliorated with USC treatment.

Our study has two limitations. First, PS/LPS-induced interstitial cystitis in rats cannot fully replicate the pathophysiological changes in humans. Second, although we found that USCs could protect the urinary bladder from IC injury in rats, the specific mechanisms of the protective effect have not been identified in the present study, and thus further studies are needed to elucidate the details of the protective mechanism.

## Conclusions

The results of this study revealed that USCs protected against PS/LPS-induced IC by suppressing oxidative stress, the inflammatory reaction, and apoptosis, and ameliorating urinary bladder environmental conditions. USCs can be accessed via a simple, noninvasive, and low-cost approach without surgical procedures. Our result may provide the basis for consideration of a prospective clinical trial to evaluate the USC clinical treatment effect of interstitial cystitis.

## Abbreviations

ADMSC: Adipose-derived mesenchymal stem cell
BPS: Bladder pain syndrome
ED: Erectile dysfunction
ER: Endoplasmic reticulum
ERS: Endoplasmic reticulum stress
HO: Heme oxygense
IC: Interstitial cystitis
IF: Immunofluorescence
IHC: Immunohistochemical
IL: Interleukin
MSC: Mesenchymal stem cell
NF: Nuclear factor
NQO: NAD(P)H quinine oxidoreductase
PBS: Phosphate-buffered saline
PS/LPS: Protamine/lipopolysaccharide
SD: Sprague–Dawley
TNF: Tumor necrosis factor
UPR: Unfolded protein response
USC: Urine-derived stem cell
